# Optimization of *in vivo* Cherenkov imaging dosimetry via spectral choices for ambient background lights and filtering

**DOI:** 10.1117/1.JBO.26.10.106003

**Published:** 2021-10-13

**Authors:** Mahbubur Rahman, Petr Bruza, Rachael Hachadorian, Daniel Alexander, Xu Cao, Rongxiao Zhang, David J. Gladstone, Brian W. Pogue

**Affiliations:** a Dartmouth College, Thayer School of Engineering, Hanover, New Hampshire, United States; b Dartmouth College, Geisel School of Medicine, Department of Radiation Oncology, Hanover, New Hampshire, United States; c Dartmouth-Hitchcock Medical Center, Norris Cotton Cancer Center, Lebanon, New Hampshire, United States; d Dartmouth College, Geisel School of Medicine, Department of Surgery, Hanover, New Hampshire, United States

**Keywords:** Cherenkov emission, ambient light, spectral filtering, patient imaging

## Abstract

**Significance:** The Cherenkov emission spectrum overlaps with that of ambient room light sources. Choice of room lighting devices dramatically affects the efficient detection of Cherenkov emission during patient treatment.

**Aim:** To determine optimal room light sources allowing Cherenkov emission imaging in normally lit radiotherapy treatment delivery rooms.

**Approach:** A variety of commercial light sources and long-pass (LP) filters were surveyed for spectral band separation from the red to near-infrared Cherenkov light emitted by tissue. Their effects on signal-to-noise ratio (SNR), Cherenkov to background signal ratio, and image artifacts were quantified by imaging irradiated tissue equivalent phantoms with an intensified time-gated CMOS camera.

**Results:** Because Cherenkov emission from tissue lies largely in the near-infrared spectrum, a controlled choice of ambient light that avoids this spectral band is ideal, along with a camera that is maximally sensitive to it. An RGB LED light source produced the best SNR out of all sources that mimic room light temperature. A 675-nm LP filter on the camera input further reduced ambient light detected (optical density > 3), achieving maximal SNR for Cherenkov emission near 40. Reduction of the room light signal reduced artifacts from specular reflection on the tissue surface and also minimized spurious Cherenkov signals from non-tissue features such as bolus.

**Conclusions:** LP filtering during image acquisition for near-infrared light in tandem with narrow band LED illuminated rooms improves image quality, trading off the loss of red wavelengths for better removal of room light in the image. This spectral filtering is also critically important to remove specular reflection in the images and allow for imaging of Cherenkov emission through clear bolus. Beyond time-gated external beam therapy systems, the spectral separation methods can be utilized for background removal for continuous treatment delivery methods including proton pencil beam scanning systems and brachytherapy.

## 1 Introduction

Cherenkov and radioluminescence imaging in recent years has been implemented into the clinic for dosimetry and positioning of patients undergoing radiotherapy. These superficial verifications can be attributed to the nearly isotropic emission in tissue and linear relationship between Cherenkov emission and dose.^1^ In external beam therapy, the imaging method was utilized for total skin electron therapy,^2^^,^^3^ head and neck,^4^ and breast patients.^5^^,^^6^ In brachytherapy of uveal melanoma, it was used to assess positioning of the irradiation source or plaque within the eye of patients.^7^ Bruza et al. imaged luminescence from biological tissue with proton pencil beam scanning (PBS).^8^ The imaging technique has been also used to characterize irradiation sources’ spatial and temporal profiles (dose profiles, source strength, position, and dwell time) for all aforementioned treatment modalities.^9^^–^^12^ Correlating dose delivery to Cherenkov emission from patients requires corrections associated with imaging perspective, anatomy, and tissue optical properties^3^ that vary from patient to patient and establishing such correction factors is well underway.^5^ Furthermore, it is worth noting that while Cherenkov light is emission in the ultraviolet and blue wavelength, emission from tissue is predominantly red and infrared weighted due to the optical absorption of water, hemoglobin, and fat.^13^

The low intensity of the emission coupled with the need of ambient room light for patient safety and comfort require consideration of the noise sources present in imaging. Although the read-out noise for cameras for a given setup (e.g., CCD or CMOS) are constant^14^^,^^15^ in comparison to other noise sources, dark current noise^16^ and photon noise^17^ can contribute significantly to the reduction in signal-to-noise ratio (SNR) and image quality of the emission profile. Furthermore, if an intensifier is included in the camera design, stray radiation from high-energy electrons can contribute further to the noise in the images.^18^ There are many algorithms utilized to improve the image quality including several median filters^19^^–^^21^ and Wiener filters.^22^^,^^23^ Nonetheless, reduction of the noise could potentially improve the image prior to implementation of any algorithm, including reduction of the photon noise by removing any ambient light that does not contribute to the desired signal. Cherenkov imaging regularly employs removal of background light to preserve image quality and enable accurate patient dosimetry.

For *in vivo* optical dosimetry of external beam radiation therapy, there have been studies on how to remove the background signal to improve the image quality. Real time Cherenkov imaging was possible by gating the camera to acquire images only during x-ray pulses and subtracting the background imaged in between pulses.^1^^,^^24^ Studies focused on selecting and evaluating the appropriate intensified cameras were conducted to further improve the image quality.^25^^,^^26^ Nonetheless, the contribution of the ambient light detected in each image can still contribute to increased noise (e.g., photon noise), reduced image quality, and artifacts. Although in external beam therapy, the time structure can be utilized for background subtraction, and this is not possible with continuous irradiation sources such as those in brachytherapy or proton PBS systems. This method is also not available for imaging Cherenkov in medical isotopes^27^ including positron emitting radiotracers examined by Robertson et al.^28^ and Spinelli et al.^29^ Investigators often resort to removing all ambient light,^7^^,^^9^^,^^12^ which is non-ideal for patient treatment scenarios.

In this study, the broadband spectral profile of Cherenkov and ambient room light were utilized to improve Cherenkov image quality. A survey of the current patient imaging set up was done, where the spectrum and illuminance of the ambient light source and Cherenkov emission were analyzed. Potential long-pass (LP) filters and commercially available ambient light sources were considered to determine, which will produce the optimal Cherenkov image quality. It was evaluated based on SNR, Cherenkov emission intensity to background ratio, and the effect of LP filter on the detected Cherenkov intensity. The spectral filtering method was also utilized to show how it can improve image quality of patients treated with optically clear bolus and reduce artifacts.

## 2 Materials and Methods

### 2.1 Patient/Experimental Imaging Setup

**Fig. 1:**
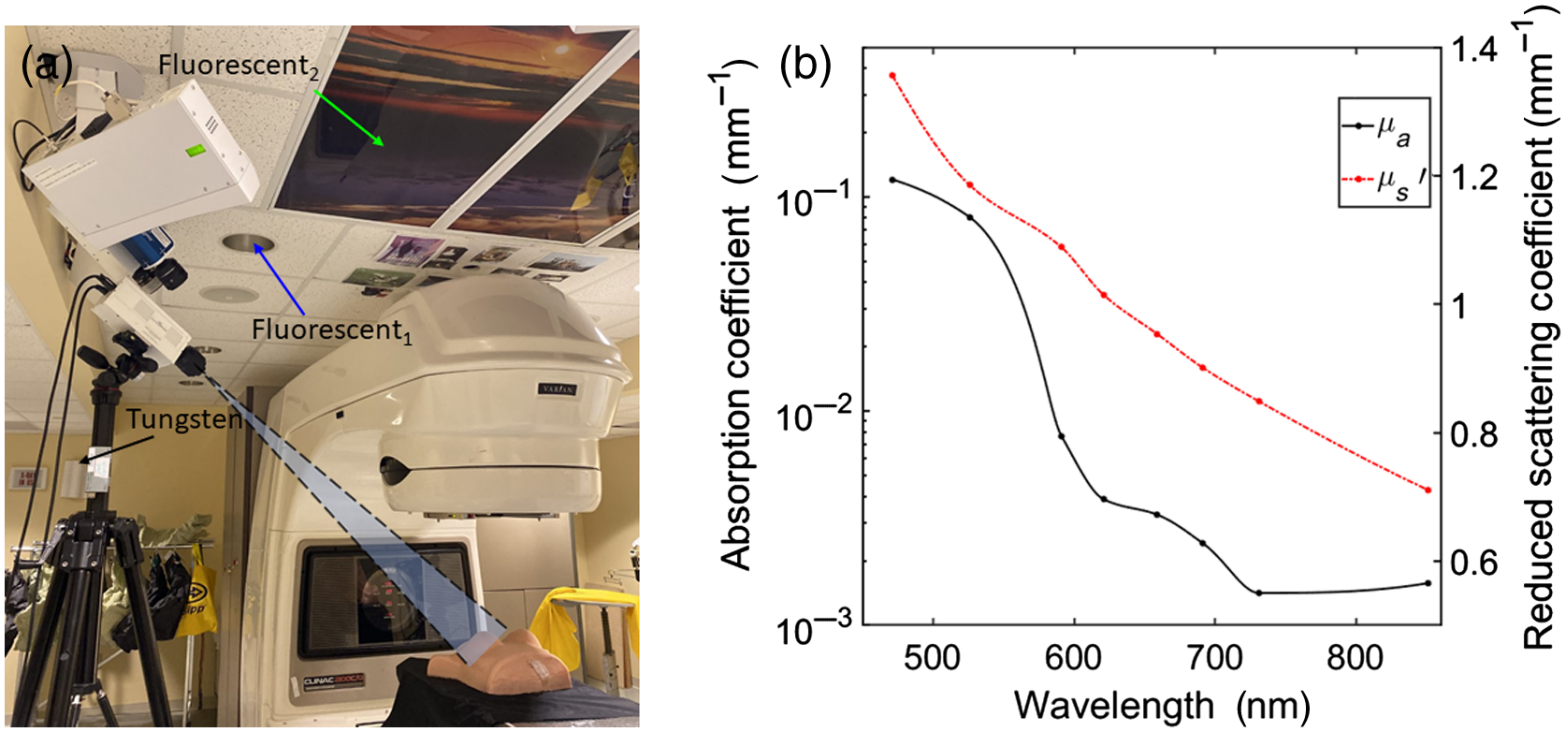
(a) Current patient imaging set up with an intensified time gated CMOS camera with an anthropomorphic breast tissue phantom as an example at isocenter and arrows indicating the current treatment room light sources. (b) Absorption coefficient ($\mu_{a}$) and reduced scattering coefficient ($\mu_{s}^{\prime }$) spectrum for the tissue phantom.^26^

**Fig. 2:**
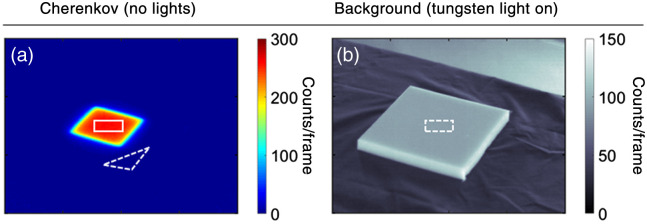
Quantifying Cherenkov emission image quality using optical filters. (a) Example Cherenkov emission image with Cherenkov (solid) and background (dashed) ROI used for calculation of SNR. (b) Example background image with (dashed) ROI used for $I_{\mathrm{CH}}/I_{\mathrm{BKG}}$ ratio.

An example set up for experimental imaging of Cherenkov luminescence from a phantom is shown in Fig. 1(a). A tripod mounted intensified CMOS camera (CDose, DoseOptics LLC, Lebanon, New Hampshire) imaged the phantom with the optical axis aligned and focused to the isocenter. A 50-mm f/1.8 lens (Nikon Inc, Tokyo, Japan) was used with a SM2 to SM1 adapter fitted with a 1-in. lens tube to attach 25-mm LP optical filters as needed (Thorlabs, Newton, New Jersey). The camera was triggered off the linear accelerator (LINAC) pulses to image during irradiation (6MV x-ray with 100 source-to-surface distance). Figure 1(a) shows an anthropomorphic tissue equivalent phantom^26^ to represent a breast treatment. A flat tissue phantom composed of the same material was also imaged at isocenter to quantify SNR and Cherenkov to room light background ratio ($I_{\mathrm{CH}}/I_{\mathrm{BKG}}$). The phantoms were representative of human tissue in that it emitted predominantly in the red and infrared wavelength due to absorption of blue and ultraviolet Cherenkov photons. Nonetheless, the phantom is homogeneous while human tissue is composed of lipids, hemoglobin, water, and melanin, each with layers and different optical properties.^30^ The optical properties (absorption and reduced scattering coefficient) of the tissue phantom are included in Fig. 1(b). The anthropomorphic phantom was used to represent and compare to typical patient geometry and imaging discussed further in Sec. 4. Cherenkov luminescence from patients was imaged with the ceiling mounted intensified CMOS camera directly above the tripod mounted camera, though both cameras have the same imaging parameters and specifications. The ambient background light sources are indicated in Fig. 1(a). The prospective light sources considered for the study were placed near the phantom covered by a diffuser to produce a homogeneous distribution of light on the phantom and held constant at 10 lux at the isocenter. An example of the Cherenkov emission and background images with a light source of the flat phantom are shown in Fig. 2.

### 2.2 Light Source and Filter Characterization

**Fig. 3:**
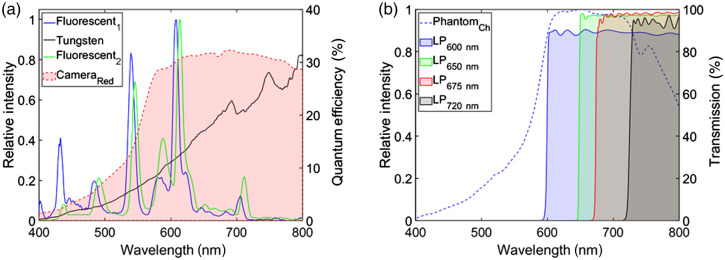
(a) Quantum efficiency spectrum of camera^4^ and ambient room light source spectrum. (b) Optical filter transmission spectrum and tissue phantom Cherenkov emission spectrum considered for improving patient imaging quality.

A spectrometer (Ocean Optics Inc., Dunedin, Florida) coupled to an optical fiber (Thorlabs Inc., Newton, New Jersey; pure silica core, 600-μm diameter, and hard polymer cladding; 0.37±0.02 numerical aperture) measured the emission spectrum of all ambient room light sources (including ones currently mounted, as shown in Fig. 3(a), and prospective ones for replacement). An intensified CCD camera (PI-MAX3, Teledyne Princeton Instruments, Trenton, New Jersey) coupled to a spectrograph (SpectraPro 2300i, Acton Research Corporation) was time gated to the LINAC pulse (at 360 Hz and 5-ms exposure time) to measure the Cherenkov emission spectrum from the tissue phantom when irradiated, as shown in Fig. 3(b). The transmission spectrum of each LP filter [Fig. 3(b)] was measured using the Varian Cary 50-Bio spectrophotometer (Agilent Technologies, Santa Clara, California). A digital lux meter (LX1330B, Dr. Meters) measured the illuminance of the light sources at the treatment room isocenter for currently mounted sources (Table 2) and prospective replacement ones (to ensure 10 lux at the isocenter).

### 2.3 Prospective Light Sources and Camera Photocathode

The types of light source, power level, color temperature, and cost were provided by each of the manufactures for the prospective replacements (included in Table 1). The lights considered were halogen lights, various LED light sources, and representative compact fluorescent and tungsten light bulbs. All the lights included in the study were commercially available and within $25 per bulb. These light sources were considered due to power levels that are typical of treatment room lights and represented a range of the color temperatures available commercially. An alternative blue sensitive photocathode for the camera was also considered to change the sensitivity spectrum of the camera and for further preferential imaging of Cherenkov emission. The Cherenkov emission was imaged with different light sources on and with or without the 675-nm LP filter. The light source was held constant at 10 lux at the isocenter and surface of the flat tissue phantom.

**Table 1:** Alternative and representative light sources considered to replace current treatment room lights (manufacturers are in Table S1 in the Supplementary Material) with specifications including power level, color temperature, and cost of each source. CFL stands for compact fluorescent light bulbs and LED stands for light emitting diodes.

Name	Watts	Color temp (K)	Cost/bulb ($)
Halogen	75	2750	17
LED amber	40	∼2000	17
LED soft white	60	2700	2
LED white	100	6000	4
LED RGB (W)	60	6000 to 6500 (2700 to 3200 K)	25
CFL	60	5000	9
Tungsten	40	2400	5

**Table 2:** Illuminance for current ambient room light sources at the treatment room isocenter.

Light source	Illuminance (lux)
${\text{Fluorescent}}_{1}$	204
Tungsten	0.6
${\text{Fluorescent}}_{2}$	51.3

### 2.4 Image Processing

**Fig. 4:**
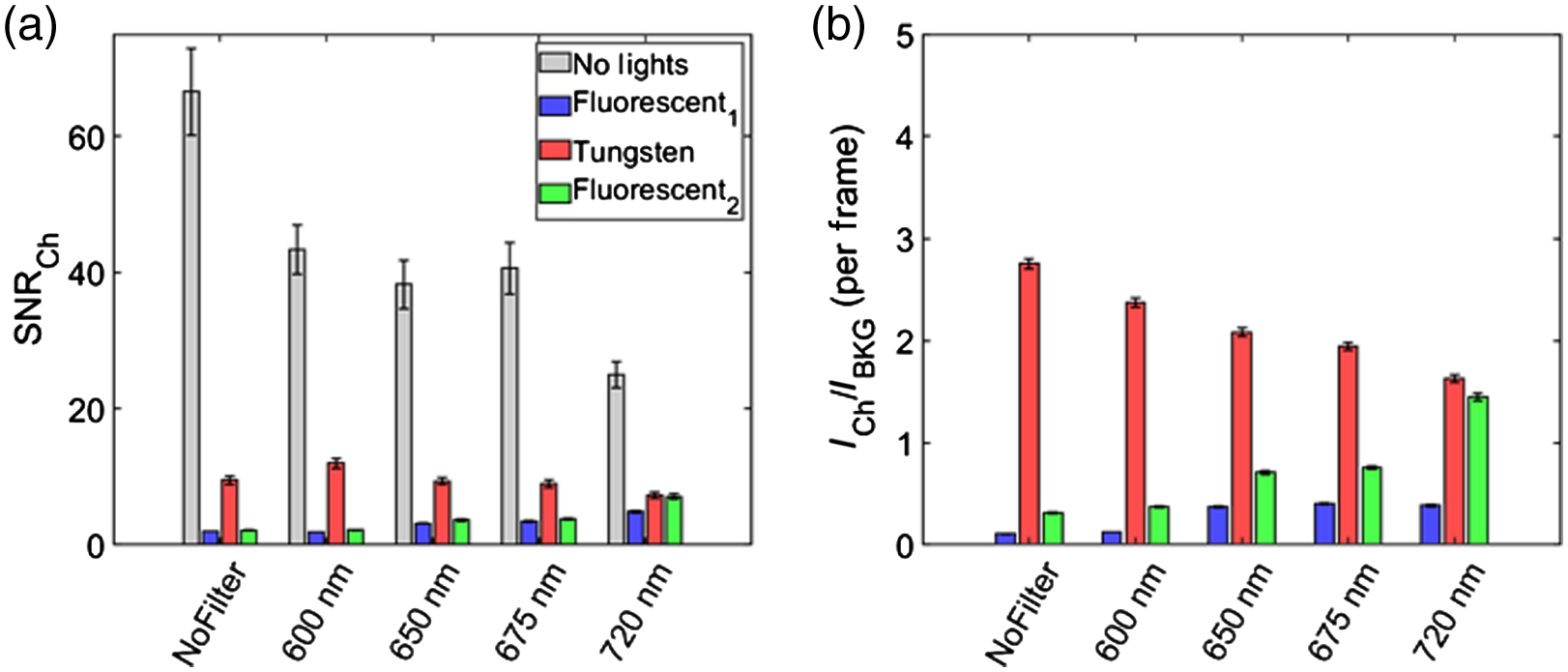
(a) SNR and (b) $I_{\mathrm{CH}}/I_{\mathrm{BKG}}$ ratio for current light sources with tested filters. (SNR and $I_{\mathrm{CH}}/I_{\mathrm{BKG}}$ ratio values are included in Table S2 in the Supplementary Material.)

**Fig. 5:**
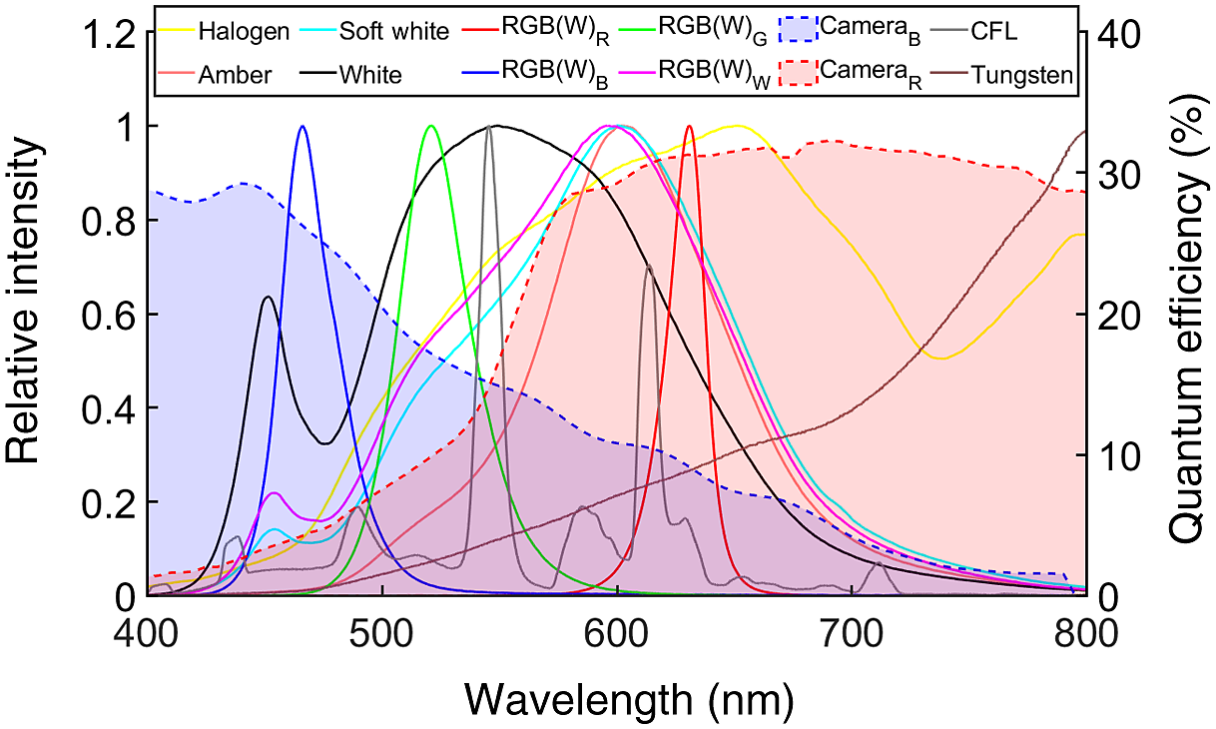
Emission spectrum of each prospective light source and quantum efficiency of considered camera photocathodes.^4^^,^^11^

**Fig. 6:**
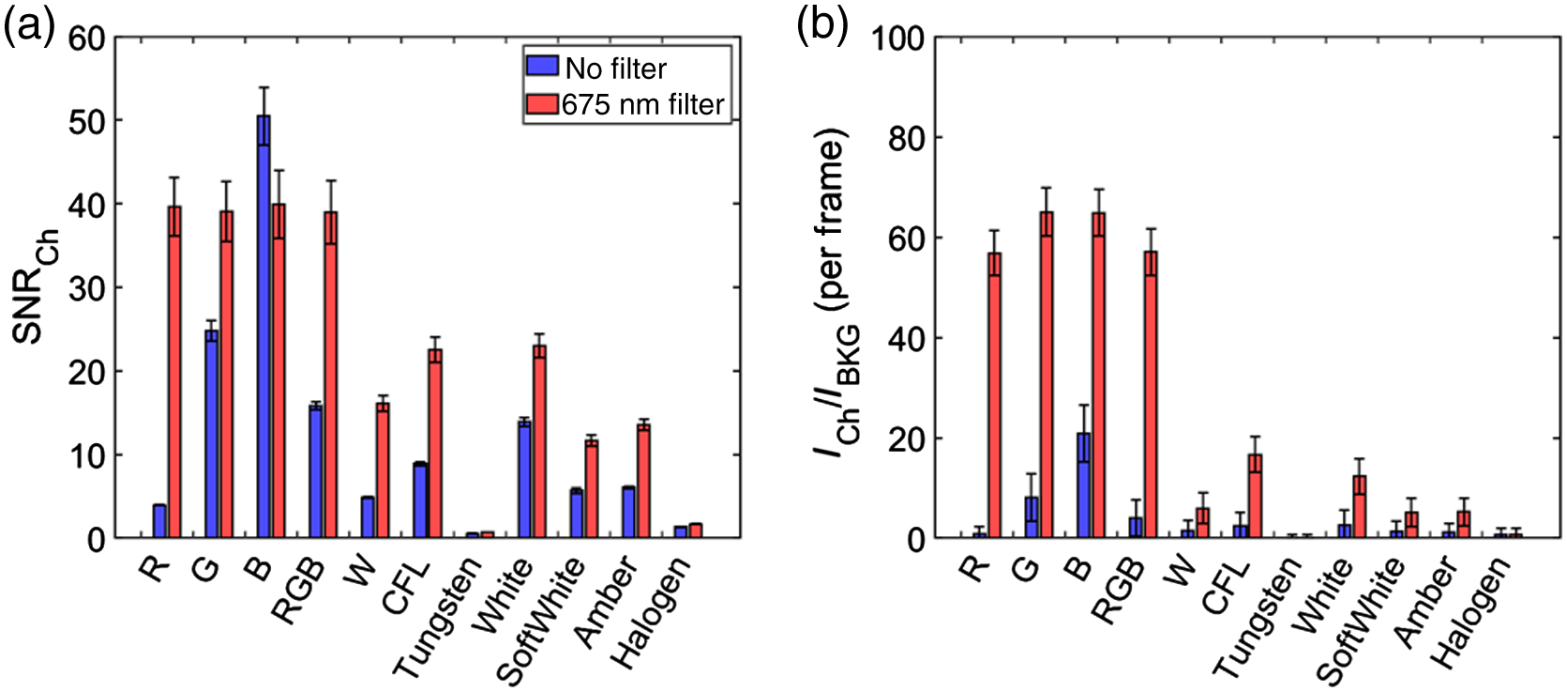
(a) SNR and (b) $I_{\mathrm{CH}}/I_{\mathrm{BKG}}$ ratio for proposed light sources considered with and without a 675-nm LP filter. (SNR and $I_{\mathrm{CH}}/I_{\mathrm{BKG}}$ ratio values are included in Table S3 in the Supplementary Material.)

**Fig. 7:**
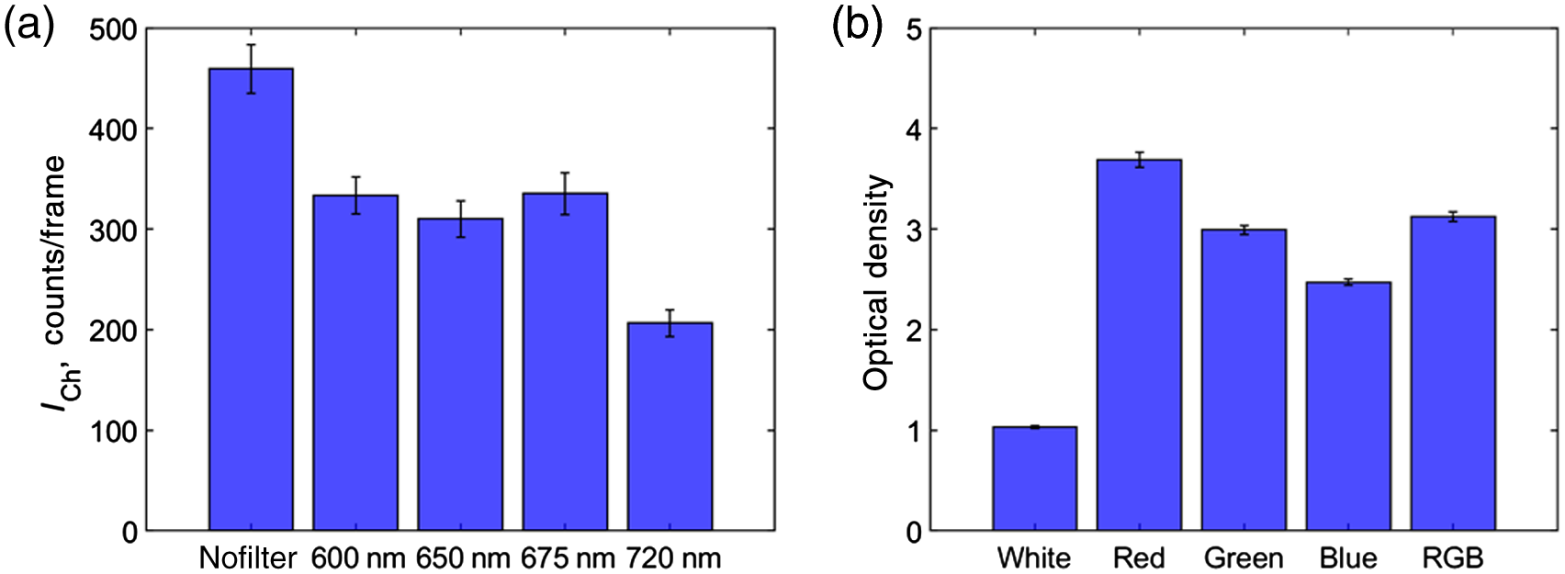
(a) Absolute Cherenkov emission count per frame with room lights off and different optical filters. (b) OD for each LED channel and RGB composite comparing with and without the 675-nm LP filter. (Method for measuring OD is included in Fig. S1 in the Supplementary Material.)

The impact of ambient light sources on Cherenkov emission image quality was quantified from both ambient room light (background) and Cherenkov images. There were 100 images per acquisition, and all had the dark signal subtracted. Each image was spatiotemporal median filtered (5×5×5  pixel window) to remove stray radiation noise.^18^ Example acquired images of the Cherenkov emission and background are shown in Figs. 2(a) and 2(b). The SNR was evaluated for each frame based on delivery of a $10\times 10\;{\mathrm{cm}}^{2}$ field from the LINAC with the ROI of the signal (rectangle) and background (triangle) shown in Fig. 2(a). The background was chosen to be on the tissue phantom but in a region with minimal Cherenkov emission from the tissue. It was defined as $\mathrm{SNR}=\text{mean}(\frac{\mathrm{CH}(i,j)-\mu_{\mathrm{BKG}}}{\sigma_{\mathrm{BKG}}})$, where i, j are the pixel coordinates, CH is the Cherenkov emission ROI, and $\mu_{\mathrm{BKG}}$, $\sigma_{\mathrm{BKG}}$ are the mean and standard deviation of the background ROI. The ratio of the intensity in Cherenkov and background light source ($I_{\mathrm{CH}}/I_{\mathrm{BKG}}$) was evaluated based on the rectangular ROI’s at the isocenter shown in Figs. 2(a) and 2(b). The current ambient sources’ SNR and $I_{\mathrm{CH}}/I_{\mathrm{BKG}}$ are included in Fig. 4. The emission spectrum of each prospective light source is included in Fig. 5, and SNR and $I_{\mathrm{CH}}/I_{\mathrm{BKG}}$ are included in Fig. 6. The absolute mean Cherenkov emission signal CH for the tested LP filters and optical density (OD) of the background signal from a 675-nm LP filter with RGB LED light source are included in Fig. 7.

### 2.5 Potential Utility of Spectral Filtering for Improved Patient Imaging

**Fig. 8:**
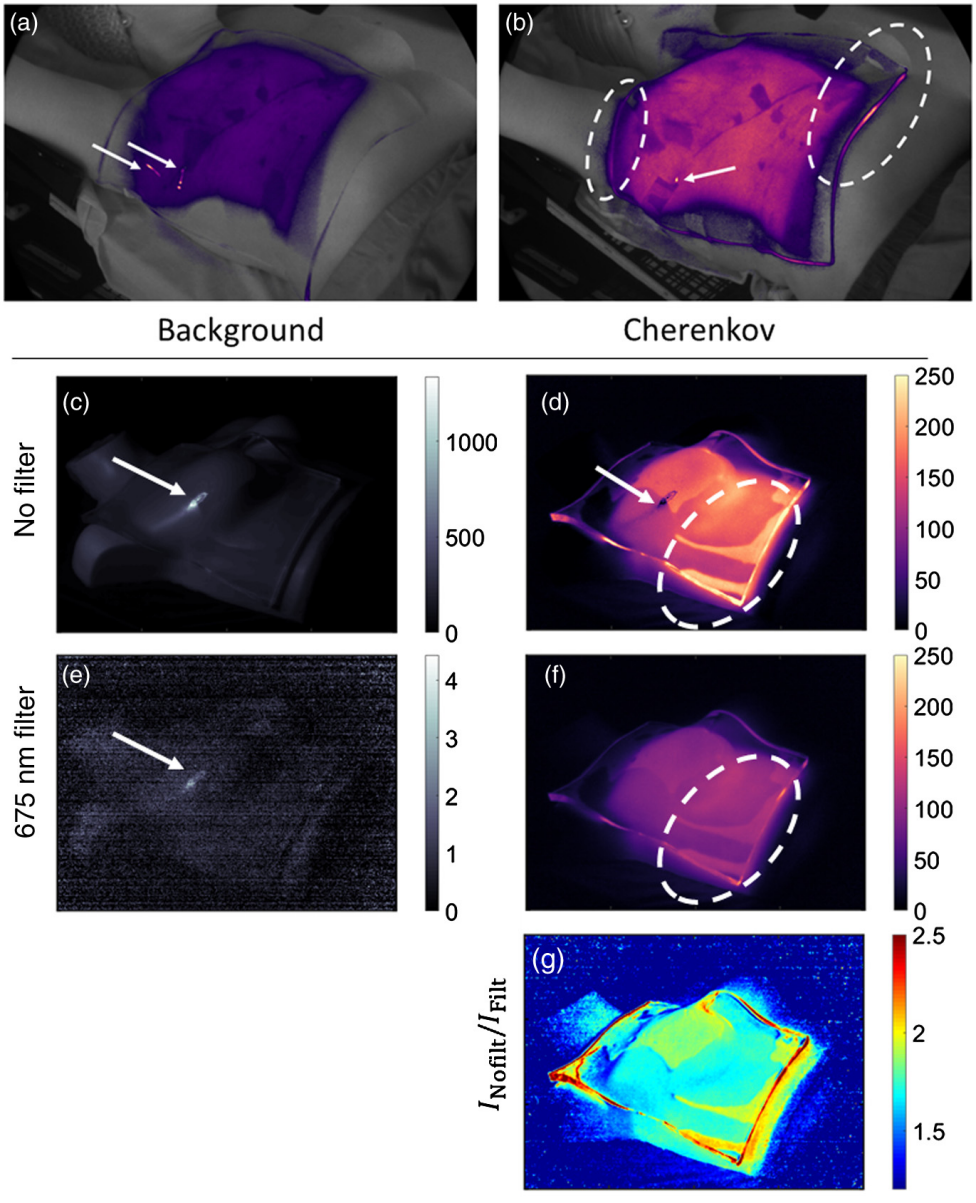
Utility of spectral background subtraction for removing image artifacts [e.g., specular reflection from ambient room light (arrows) and total internal reflection of light within transparent bolus (dotted ovals)]. (a), (b) Example Cherenkov emission acquisition from a patient treated with bolus. (c)–(g) Effects of 675-nm LP optical filter on image artifacts with RGB LED light source.

The anthropomorphic phantom was imaged with an optical filter and ambient room light source combination that produced the best conditions for patient imaging (considering room light temperature, SNR, and $I_{\mathrm{CH}}/I_{\mathrm{BKG}}$ ratio) to illustrate a potential utility of spectral filtering (Fig. 8). Representative of a patient treated with an optically clear bolus [Figs. 8(a) and 8(b)], a 1-cm bolus was placed on top of the right breast of the phantom and the breast was irradiated with a $20\times 20\;{\mathrm{cm}}^{2}$ field. The camera imaged the background and Cherenkov emission of the phantom with or without the optical filter [Figs. 8(c)–8(f)]. The ratio of the Cherenkov images was determined dividing the emission profile with and without the optical filter [$I_{\text{NoFilt}}/I_{\text{Filt}}$, Fig. 8(g)]. For comparison, the phantom was also imaged with the current room light source (tungsten) and with or without the optical filter, which is included in Fig. S2 in the Supplementary Material.

## 3 Results

### 3.1 Current Patient Imaging Set up

The current patient imaging set up captures a non-negligible superposition of both ambient room light and Cherenkov emission from the patient surface, as shown in Figs. 3(a) and 3(b). The tissue equivalent phantom, representative of patients, emitted predominantly above 600 nm, which was appropriately imaged with a photocathode sensitive to photons above 560 nm (quantum efficiency above 20%). Nonetheless, the ambient light sources exhibited a broad emission spectrum as well. The fluorescent light sources have similar emission spectrum with peaks ranging from 400 to 710 nm, with the largest peak at 607 and 614 nm for fluorescent sources 1 and 2, respectively. The two fluorescent sources had a much higher light level of 204 and 51.3 lux in comparison to the tungsten source of 0.6 lux. Though dim, the tungsten light source emitted predominantly above 600 nm and in the near-infrared (NIR), which is comparable to the Cherenkov emission spectrum of the tissue phantom.

### 3.2 Spectral Filtering

LP spectral filters attached to the camera were used to evaluate their ability to suppress ambient light while maintaining image quality of Cherenkov emission surface profiles. All filters suppressed light with an OD>3 below the cut on wavelength specified in Fig. 3(b). While an ideal 600-nm LP filter would allow the most photons to be detected (in comparison to the other filters), the spectrum shows the 600-nm LP filter transmits ∼90% of the light above its cut on wavelength, but the other filters (650, 675, and 720 nm) transmit ∼95% or more of light above its cut on wavelength. Nonetheless, the SNR and $I_{\mathrm{CH}}/I_{\mathrm{BKG}}$ ratio for each of the filters and with each light source on are shown in Figs. 4(a) and 4(b). As expected, when no room lights were on, spectral filtering simply reduced Cherenkov emission signal and the SNR with the greatest reduction was the 720-nm filter (from SNR of 67±6 to 25±2 per frame). The 600-, 650-, and 675-nm LP filters had comparable SNR of ∼40 (43±4, 38±4, 41±4, respectively).

When room lights were on, the spectral filters had contrasting results for the tungsten light sources and the fluorescent sources. The SNR for fluorescent 1 light source was less than fluorescent 2 while having similar spectrums, which can be attributed to the greater illuminance (fluorescent 1 was brighter). However, spectral filtering up to 720 nm for both fluorescent sources improved the SNR from 1.95±0.02 to 4.8±0.2 and 2.14±0.05 to 7.1±0.4, respectively. The $I_{\mathrm{CH}}/I_{\mathrm{BKG}}$ ratio for the two fluorescent sources improved by a factor of ˜4. However, the SNR was comparable and even reduced when using the filters with the tungsten light source. There was a reduction of the $I_{\mathrm{CH}}/I_{\mathrm{BKG}}$ ratio by about 70%. Although SNR did improve with the fluorescent sources and the 720-nm filter, it is worth noting that the Cherenkov emission signal was reduced by a factor of ∼2.3 (shown in Fig. S1 in the Supplementary Material).

### 3.3 Light Source Replacements

From the spectra shown in Fig. 5, most of the light sources considered have very broad spectra, including the LED lights. The compact fluorescent light (CFL) and tungsten light sources had very similar spectra to the ones that are currently installed. The halogen bulbs emitted in a broad spectrum above 500 nm that extended to the NIR. The amber, soft white, and white LED though emitted in a broad spectrum, predominately emitted below 700 nm. The RGB(W) LED light source exhibited the greatest potential for replacing the current light sources due to narrow peaks for the red, green, and blue source (though the white source in the RGB(W) was similar to the soft white). A blue sensitive photocathode was also considered, but it was least sensitive at the wavelengths of Cherenkov radiation emitted from tissue.

As shown in Fig. 6, the highest SNR and $I_{\mathrm{CH}}/I_{\mathrm{BKG}}$ ratio were achieved by the blue channel on the RGB(W) LED light source, which can be attributed to the emission peak (465 nm) being in the spectrum where the red photocathode is the least sensitive. The tungsten and halogen bulbs resulted in the least SNR and $I_{\mathrm{CH}}/I_{\mathrm{BKG}}$ ratio, which can be attributed to the broad emission spectrum weighted toward red and NIR wavelengths. Nonetheless, the 675-nm LP filters imaged Cherenkov emission showed a great improvement in the SNR and $I_{\mathrm{CH}}/I_{\mathrm{BKG}}$ ratio, particularly for the red (R), green (G), and blue (B) light sources due to their narrow peaks. Imaging with any of these sources and their composite with the 675-nm LP filters showed comparable SNR and $I_{\mathrm{CH}}/I_{\mathrm{BKG}}$ ratio. This can be attributed to the LP filter’s ability to suppress the RGB LED light source with an OD of 3.13±0.05 [shown in Fig. 7(b)]. The optical filter also improved the image quality for other LED light sources (white, soft white, and amber), halogen, and CFL because the filter suppressed a large fraction of the light emitted from the sources below 675 nm while maintaining ∼70% of the Cherenkov emission detected by the camera [Fig. 7(a)].

### 3.4 Potential Utility of Spectral Filtering for Improved Patient Imaging

Figure 8 shows a potential utility of the spectral filtering in a current image setup with optically clear bolus. Imaging patients with room lights on presented specular reflection [indicated with arrows in Figs. 8(a) and 8(b)], which can make it difficult to correlate Cherenkov emission to surface dose through the bolus.^5^ Furthermore, the light piping of optically clear Cherenkov emitters such as the bolus would make it difficult to quantify dose at the edge of the bolus or treatment fields [indicated with dashed ovals in Fig. 8(b)]. Figures 8(c) and 8(e) show results with narrow band RGB LED light sources and a 675-nm LP filter; the specular reflection was negligible as the ambient light detected was reduced by an OD>3. Figure S2 in the Supplementary Material shows the specular reflections were prominent in the Cherenkov emission profile with the tungsten light source, both with and without the LP filter. Figure 8(g) shows that Cherenkov emission from optically clear bolus was disproportionately suppressed in comparison to the tissue phantom, as the edges of the bolus with the light piping effect showed the $I_{\text{NoFilt}}/I_{\text{Filt}}∼2.5$, and the surface most orthogonal to the camera’s optical axis and surfaces without bolus had $I_{\text{NoFilt}}/I_{\text{Filt}}∼1.7$.

## 4 Discussion

While keeping in mind the overall goal of improving Cherenkov images with minimal changes to the normal treatment delivery conditions (including ambient room light), some factors considered when choosing the light source and optical filter included color temperature, illuminance, emission spectrum, and the amount of Cherenkov signal suppressed. The current room light sources in the treatment room reduced the SNR of the Cherenkov images below 10. For the fluorescent light sources, this can be attributed to the illuminance level being above 50 for both at the treatment room isocenter. Although the tungsten light sources have a very low illuminance of 0.6, this source can still reduce SNR due to the broad spectrum of light emission in the red and NIR region. Such low-light levels are also non-ideal for patients because typical illuminance of rooms is above 10 lux. The blue channel of the RGB (W) light source considered as a replacement shown in Fig. 6 would be the best alternative to the current room light sources, because it exhibited the best SNR given that the camera predominantly detected photons above ∼560  nm. However, the color temperature (∼10,000  K) was much higher than the typical 2500 to 6500 K found in room lights. A superposition of the RGB channel light sources with a 675-nm filter was the best alternative to imaging with ambient room light because the optical filter suppressed the room light to below 2% of the Cherenkov signal, with a color temperature of 6000 to 6500 K.

As shown in Fig. 8, spectral filtering can have particular utility in imaging patients with the narrow band light sources (e.g., RGB LED). Beyond suppressing ambient light sources for improved SNR, the light piping artifact from optical clear material such as bolus can be reduced due its blue weighted Cherenkov emissions.^4^ This can potentially help in imaging and quantification of radiation dose^3^^,^^5^ and treatment field verification^31^ of the tissue underneath, which emits red light^32^ and to which the photocathode used for imaging was sensitive. The filtering can suppress ambient light optical artifacts such as specular reflection to be negligible [Figs. 8(c)–8(f)]. The light source choice is important to specular reflection suppression, as indicated by Fig. S2 in the Supplementary Material, since the spectral filtering could not remove the reflections produced from the currently mounted tungsten light sources. Nonetheless, there may also be specular reflection from patients’ perspiration and moist desquamation, and it would be worth investigating the effect of optical filtering on the Cherenkov profile from those sources of artifacts in the future.

Spectral filtering may also find utility in surface guided radiation therapy (SGRT) when used in conjunction with Cherenkov emission imaging. SGRT often utilizes optical surface imaging techniques to track patient motion and ensure consistent patient set up with commercially available product including align RT (Vision RT, London, United Kingdom), catalyst (C-Rad, Upsalla, Sweden), and identify (Varian Medical System, Inc., USA).^33^ The products utilize LED light sources projected onto the patient for position tracking, which can potentially be suppressed via filtering to image the Cherenkov emission during irradiation. For example, the catalyst emits light sources with peak wavelengths of 405, 528, and 624 nm^34^ suggesting a majority of the projected light signal would be suppressed with the 675-nm LP filter considered in this study. Nonetheless, the extent of signal suppression for each of the optical surface imaging devices and their effect on Cherenkov emission imaging require further investigation.

Although narrow band LED bulbs can be spectrally suppressed for improved luminescence image quality, one potential shortcoming can be the light source flicker as Cherenkov imaging typically relies on background subtraction from images. Without an optical filter, if the repetition rate of the external beam treatment delivery system does not match the pulse width modulation of the LED bulbs, the camera gated to the LINAC pulse^10^^,^^24^ will observe variable background in the frames. This can be solved with a constant-current LED driver to modulate in the intensity, instead of the frequency domain. Compact fluorescent bulb even though exhibiting a repetition rate was not observed to produce such a flicker, so may be an alternative to using the LED bulbs if background frames of patients are desired and optical filtering is not possible.

Nonetheless, spectral filtering of ambient room light can potentially improve patient imaging of luminescence for other treatment modalities, particularly those for which a camera cannot be time gated. Yabe et al.^12^ showed that Cherenkov emission from proton beam irradiation can be utilized to characterize the beam for PBS systems. Bruza et al.^8^ imaged the light emission from biological tissue irradiated with a PBS system. PBS systems deliver dose continuously through spot scanning and the spectral removal of the ambient light can be a viable method to ensure room light for patient while imaging their Cherenkov profile from irradiation. Cherenkov luminescence imaging was also utilized for quality assurance of brachytherapy radiation sources,^9^ radiotracers,^27^^–^^29^ and *in vivo* imaging for treatment of uveal melanoma.^7^ However, the ambient room light was suppressed using black curtains or by turning off the room lights, which are both not ideal for luminescence imaging of patients. Thus the spectral filtering of ambient room light can provide an alternative to suppressing the background light to maintain patient comfort and image quality.

## 5 Conclusion

In this study, spectral filtering of the ambient room light improved the image quality of Cherenkov profiles. The best images were produced using a 675-nm LP filter with RGB LED lighting that the best represented current treatment room ambient room lighting. This was possible due to the narrow emission wavelength bands of the chosen LEDs. Although the filtering results in imaging Cherenkov emission predominantly in the NIR region and reduction in its intensity by ∼30%, the reduction in noise from the background sufficed in improving the SNR and image quality. The filter method disproportionately suppressed Cherenkov emission from optically clear medium (i.e., bolus), and ambient light to remove artifacts that could potentially improve surface dosimetry via Cherenkov patient imaging in the future studies. This method will be particularly useful for temporally continuous irradiation modalities such as brachytherapy or proton beams.
