# Supplementary material for: Optimization of in vivo Cherenkov imaging dosimetry via spectral choices for ambient background lights and filtering
Source: J Biomed Opt. 2021 Oct 13;26(10):106003. doi: 10.1117/1.JBO.26.10.106003 (PMC8510878; doi:10.1117/1.JBO.26.10.106003)
Supplement: Supplementary file 1 [file JBO_026_106003_SD001.pdf]

Supplementary Material A

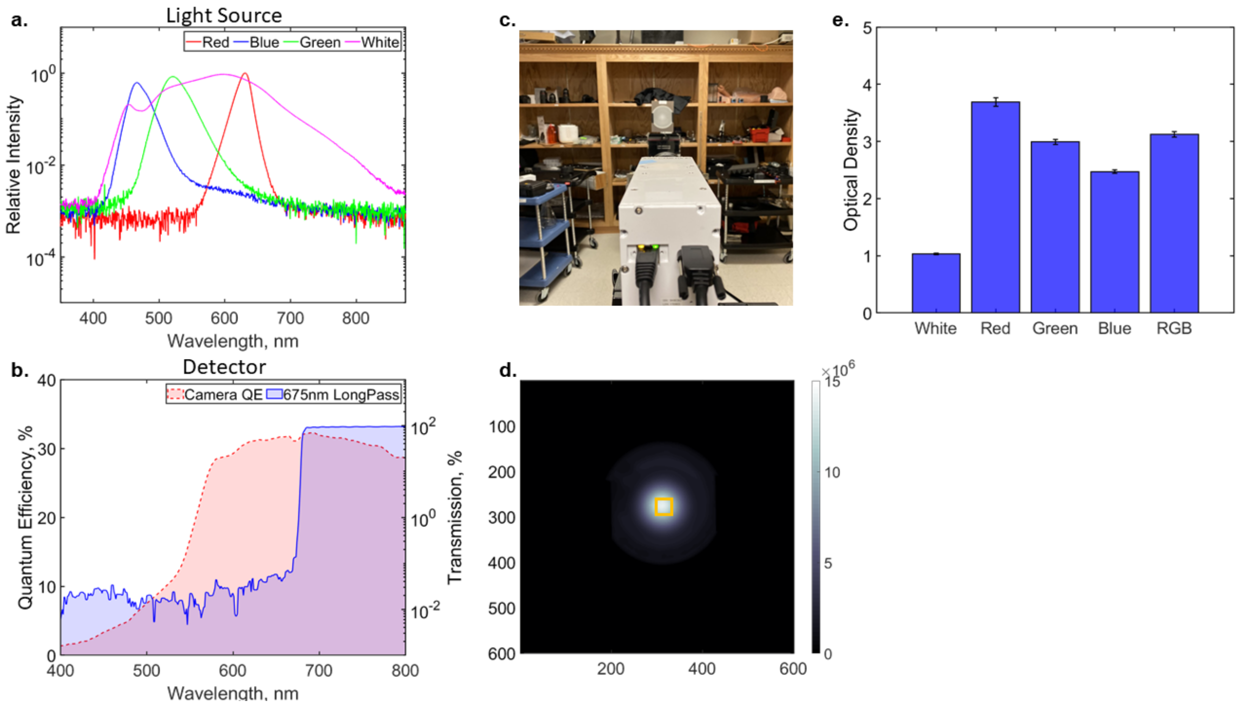

Supplementary Figure A1. Determining optical density (OD) with an RGB(W) LED light source and 675 nm long pass filter. **a.** emission spectrum of RGB (W) LED in log scale **b.** quantum efficiency spectrum of camera and transmission spectrum of 675 nm long pass filter in log scale. **c.** Camera set up with a diffuser over the light source **d.** Example acquired image with an ROI taken at the center of the image **e.** Optical density for each LED channel and RGB composite comparing with and without the long pass filter.

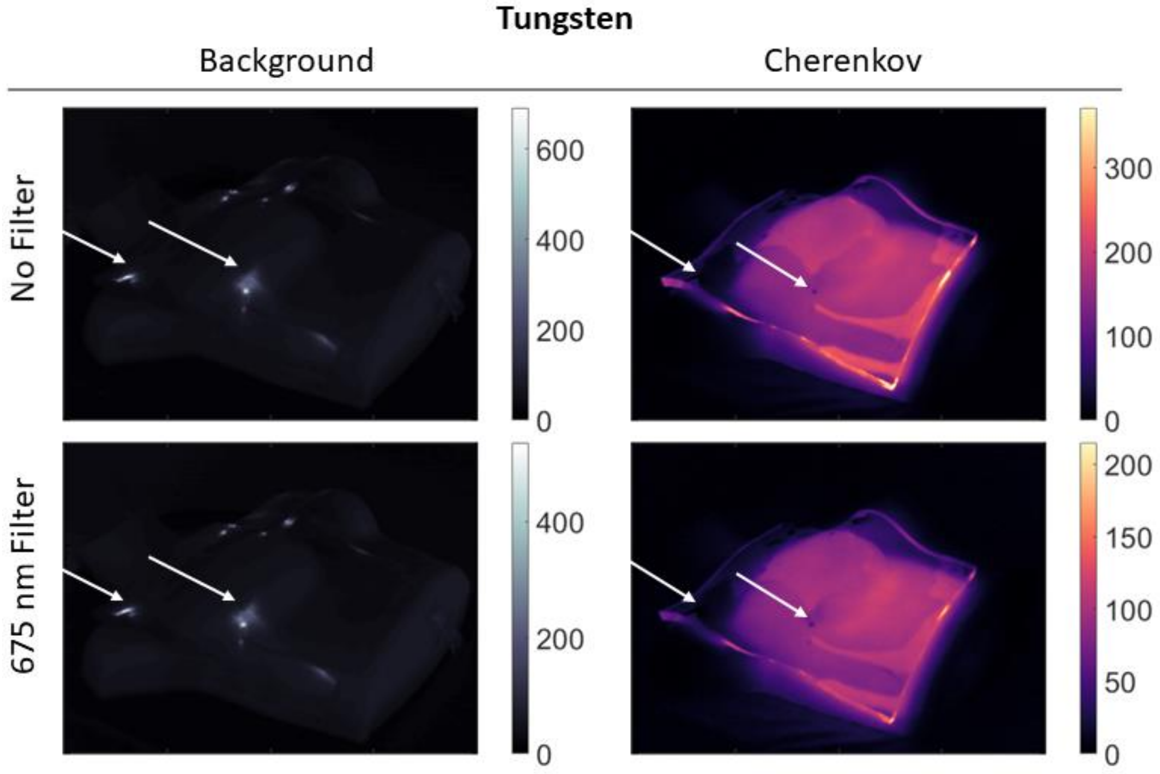

Supplementary Figure A2. Effects of 675 nm long pass optical filter on image artifacts with tungsten light source

Light Source	Manufacturer/Product
Halogen	General Electric/HIR Plus XL
LED Amber	Harth/ Nite-Nite
LED Soft White	Ecosmart/ Soft White
LED White	Glen Mila/ GM-A19-E26-11
LED RGB (W)	MagicLight/ Smart LED Bulb
CFL	Ecosmart/ Daylight GP19 CFL
Tungsten	Feit Electric/Amber Glass ST19

Supplementary Table 1. Manufacturers of the alternative and representative light sources considered to replace current treatment room lights.

a.

Ambient Light Source\Optical Filter	No Filter	600 nm LP	650 nm LP	657 nm LP	720 nm LP
No Light	66±6	43±4	38±4	41±4	25±2
Fluorescent 1	1.95±0.02	1.85±0.03	3.15±0.08	3.4±0.1	4.9±0.2
Tungsten	9.5±0.7	12.0±0.7	9.3±0.5	8.9±0.6	7.3±0.4
Fluorescent 2	2.14±0.05	2.19±0.06	3.6±0.1	3.8±0.1	7.1±0.4

b.

Ambient Light Source\Optical Filter	No Filter	600 nm LP	650 nm LP	657 nm LP	720 nm LP
Fluorescent 1	0.313±0.006	0.369±0.008	0.71±0.01	0.75±0.02	1.44±0.04
Tungsten	2.75±0.05	2.37±0.05	2.08±0.04	1.94±0.04	1.62±0.04
Fluorescent 2	0.105±0.003	0.124±0.003	0.371±0.009	0.400±0.009	0.39±0.01

Supplementary Table 2. SNR (**a.**) and I_{CH}/I_{BKG} ratio (**b.**) for current light sources with tested filters.

a.

Ambient Light Source\Optical Filter	No Filter	675 nm LP
R LED	3.95±0.06	40±4
G LED	25±1	39±4
B LED	50±3	40±4
RGB LED	15.8±0.5	39±4
W LED	4.83±0.08	16.1±0.9
CFL	8.9±0.3	23±2
Tungsten	0.57±0.01	0.73±0.02
White LED	13.9±0.5	23±1
Soft White LED	5.67±0.3	11.6±0.6
Amber LED	6.0±0.1	13.5±0.6
Halogen	1.32±0.02	1.67±0.04

b.

Ambient Light Source\Optical Filter	No Filter	675 nm LP
R LED	0.80±0.04	57±10
G LED	8.1±0.4	65±12
B LED	21±1	65±16
RGB LED	4.0±0.1	57±11
W LED	1.44±0.04	6.0±0.2
CFL	2.45±0.06	17±1
Tungsten	0.188±0.006	0.135±0.004

White LED	2.59±0.06	12.3±0.6
Soft White LED	1.41±0.03	5.2±0.2
Amber LED	1.11±0.03	5.3±0.2
Halogen	0.67±0.02	0.77±0.02

Supplementary Table 3. SNR (**a.**) and I_{CH}/I_{BKG} ratio (**b.**) for proposed light sources with and without 675 nm LP filter.
